# Genetic association between the *Pfk13* gene mutation and artemisinin resistance phenotype in *Plasmodium falciparum* isolates from Yunnan Province, China

**DOI:** 10.1186/s12936-018-2619-4

**Published:** 2018-12-18

**Authors:** Ying Dong, Jian Wang, Aiming Sun, Yan Deng, Mengni Chen, Yanchun Xu, Jingpo Xue

**Affiliations:** 1 Yunnan Institute of Parasitic Diseases Control, Yunnan Provincial Key Laboratory, Yunnan Centre of Malaria Research, Pu’er, 665000 China; 2 Hubei International Travel Healthcare Centre, Wuhan, 430000 China; 3 National Institute of Parasitic Diseases, Chinese Center for Disease Control and Prevention, Shanghai, 200025 China

**Keywords:** Yunnan, Myanmar, Falciparum malaria cases, *PfK13* gene, Artemisinin resistance, Associative analysis

## Abstract

**Background:**

The problem of anti-malarial drug resistance is a long-term challenge faced by malaria control in Yunnan Province. Recently, the detection rates of chloroquine-resistant molecular markers (*Plasmodium falciparum* chloroquine resistant transporter, *Pfcrt*) and artemisinin-resistant molecular markers (*P. falciparum kelch13* gene, *ork13*) were 85% and 35%, respectively. To understand the association of *k13* gene mutation with artemisinin resistance in falciparum malaria cases, the difference in *k13* gene differentiation between two populations and artemisinin resistance phenotype on falciparum malaria cases in Myanmar were analysed in this study.

**Methods:**

This research involved all of falciparum malaria cases diagnosed continuously in Yunnan Province from 2013 to 2015 and some of falciparum malaria cases found in Lazar, Myanmar. Blood samples were taken from the former group for molecular epidemiological analysis of *k13* gene mutations, and artemisinin resistance phenotypes of *P. falciparum* were observed in the latter group using the in vivo testing method recommended by the World Health Organization. Nested PCR was used to amplify the propeller domain of the *k13* gene in *P. falciparum*, followed by sequencing.

**Results:**

A total of 202 blood samples were collected from Yunnan Province and 382 blood samples were collected from falciparum malaria cases in Myanmar. 49 of 382 Myanmar cases were in vivo tested for artesunate resistance phenotype through full treatment course observation. At the same time, all the blood samples were screened for *k13* gene mutation of *P. falciparum.* The genetic diversity of *k13* was higher in the *Plasmodium* isolates from Yunnan Province than those from Myanmar cases. The genetic differentiation index of the two populations was 0.0410, where the intra- and inter-group variations were 95.9% and 4.1%, respectively. The odds ratio of artemisinin resistance phenotype and mutation at the locus 446 in *k13* gene in Myanmar cases was 1.640, while the value was 1.840 based on the estimations of the mutations in the 12 loci.

**Conclusion:**

Although the *Plasmodium* isolates from Yunnan Province and those from Myanmar were collected from different sites, they still belong to the same geographical population. It is, therefore, reasonable to contrast the artemisinin resistance status of the *Plasmodium* population from Myanmar with the *Plasmodium* population from Yunnan Province. As a result, based on the molecular epidemiological investigation on *k13* mutations of *Plasmodium* isolates in Yunnan Province and the determination of the artemisinin resistance on falciparum malaria cases in Myanmar, the positively genetic correlated was found between the *k13* locus mutations with artemisinin resistance phenotype. This provides a basis for further monitoring the artemisinin resistance by detection some molecular markers in *k13* gene of *Plasmodium* in Yunnan Province.

## Background

Artemisinin is a sesquiterpene lactone, containing the peroxide group, extracted and isolated from the leaves of *Artemisia annua.* The drug and its derivatives play a role in killing *Plasmodium falciparum* by inhibiting the activity of phosphatidylinositol-3-kinase (PfPI3K) [1], with few side effects [2, 3]. Therefore, the World Health Organization (WHO) has advocated artemisinin-based combination therapy (ACT) as the first-line anti-malarial treatment of uncomplicated falciparum malaria in malaria-endemic areas to effectively reduce the incidence of the disease and the risk of death, thereby significantly reducing the burden of malaria worldwide [4]. The first discovery of artemisinin-resistant isolates in Cambodia in 2008 [5], was followed by a spread to Myanmar and Thailand [5–10]. Presently, artemisinin resistance is primarily observed in Cambodia, Laos, Thailand, Myanmar, and the shared border with Yunnan Province, China [11–14].

The clinical resistance phenotype of *P. falciparum* to artemisinin is a prolongation of *Plasmodium* clearance time in human circulating blood [6, 7]. Consequently, the expression of PfPI3K is up-regulated in artemisinin-resistant parasites [1]. Mbengue et al. [15] further confirmed that some loci mutations in the *k13* gene in *P. falciparum* could lead to this altered expression. In Cambodia [16], Southeast Asia [17], and other areas, the prolonged duration for *Plasmodium* clearance is associated with the propeller domain loci mutations in the *k13* gene of *P. falciparum* isolates. Adams et al. [18] demonstrated that the propeller area of the *Pf*K13 protein is associated with a variety of cellular functions, such as ubiquitin-regulating proteins and oxidative stress, and the loci mutations in that area may alter the interaction of these proteins. Although Tun et al. [19], Wang et al. [20], and Huang et al. [21] have found that the F446I locus mutation in the propeller domain of the *k13* gene in *P. falciparum* isolates in Myanmar has a stable impact on the artemisinin resistance appearance, the genetic relationship within different populations is yet to be elucidated.

In the early 1980s, the chloroquine resistance of *P. falciparum* was monitored systematically in Yunnan Province [22–26]. Based on the clinical curative effect [27–29], artemisinin was used gradually for the treatment of cerebral malaria and chloroquine-resistant falciparum malaria in malaria-endemic areas [30–32]. In 1996, a decreased sensitivity to artemisinin was detected in the treatment of falciparum malaria in Yunnan [33]. By 2005, the rate of chloroquine resistance in the main falciparum malaria endemic areas of the province was about 70% [34], while the minimum inhibitory concentration of artemisinin increased four- to eightfold [35]. Recently, molecular markers of *P. falciparum* chloroquine resistance (*Pfcrt* gene) and artemisinin resistance (*kelch13* gene) were monitored. The results show that mutations of *Pfcrt* gene were identified from 81.3% isolates in Yunnan Province [14], and the detection rates of chloroquine-resistant *Pfcrt* and artemisinin-resistant *k13 P. falciparum*, were 85% and 35%, respectively [36, 37]. The isolates having the two resistant molecules accounted for about 27.1% of the population [38], demonstrating the complexity of drug resistance of *P. falciparum* in Yunnan Province.

However, genetic markers alone might not be sufficient for determining the status of anti-malarial resistance in a region, and hence, it is essential to observe the resistance phenotype in a specific number of *P. falciparum* samples. In recent years, the number of Yunnan indigenous falciparum malaria cases has declined to less than 5 per year, along with the cases that meet the conditions of in vivo test for *Plasmodium* resistance [39]. In contrast, the prevalence of falciparum malaria is still serious in Myanmar, which borders Yunnan Province. More than 70% cases diagnosed and reported by Yunnan Province are still infected ‘in the region’, rather than indigenous infection [37].

Therefore, understanding the artemisinin-resistant phenotype of falciparum malaria cases in Myanmar not only facilitates the selecting of a reasonable scheme for standardized treatment of the falciparum malaria cases infected in Myanmar, but also may provide a solution to the predicament that in vivo testing anti-malarial drug resistance are unable to be carry out in Yunnan Province due to the lack of indigenous infection volunteers. If the genetic similarity of *Plasmodium* isolates between Yunnan Province and Myanmar could be proved enough high, it would represent that the biological characteristics of the two groups isolates are stable homogeneity, and the artemisinin resistance characteristics observed from one group could be regarded as a common feature of both groups. Consequently, in this study the association between the mutation of *k13* and artemisinin resistance of *Plasmodium* isolates from Yunnan Province was analysed by using population genetics analytic method, while the artemisinin resistant phenotypes of *P. falciparum* had to be tested in vivo on falciparum malaria cases in Myanmar.

## Methods

### Ethics statement

The study was approved by Yunnan Institute of Parasitic Diseases and by the Ethical Committee. Genetic testing was performed on stored blood samples obtained as part of routine diagnostic work-up patients with fever suspected of malaria. Although the absence of risk and the anonymous data processing, during collecting samples suspected of malaria person need to obtain informed consent.

### Subjects and blood sample collection

This research involved all of falciparum malaria cases diagnosed continuously in Yunnan Province from 2013 to 2015 and parties of falciparum malaria cases found in Lazar, Myanmar. Molecular epidemiological analysis of *k13* gene mutation in *P. falciparum* from the former group case blood samples was carried out, and the artemisinin resistance phenotype of *P. falciparum* was mainly observed in the latter group cases.

### Malaria cases in Yunnan Province

The falciparum malaria cases were diagnosed by the health and medical institutions in 16 prefectures and 129 counties according to the Diagnostic Criteria for Malaria (WS259-2006) [40], and confirmed by the malaria diagnosis reference laboratory using genetic test [41]. Consecutively, these cases were registered in the China Information System for Disease Control and Prevention, who belonged to the cases recognized and reported by officially, and could be classified into Yunnan indigenous infection cases and infection cases imported mainly from Myanmar and Africa. The nature of these cases was determined by epidemiological surveys, i.e., cases of patients who did not travel outside Yunnan 30 days before the onset of malaria were defined as indigenous cases, while those who travelled to Myanmar or African countries were defined as Myanmar and African infection cases, respectively.

### Malaria cases in Myanmar

These malaria cases with only infection *P. falciparum* were diagnosed by microscopy and genetic testing, and the patients were found to be settled for long-term in Lazar of Myanmar, which borders Yunnan Province, China [42]. These cases included all patients irrespective of their participation in the genetic relationship studies, aged 2–60 years, were not administered anti-malarial and/or antibacterial drugs within 2 weeks, and underwent artesunate resistance in vivo test.

**Fig. 1:**
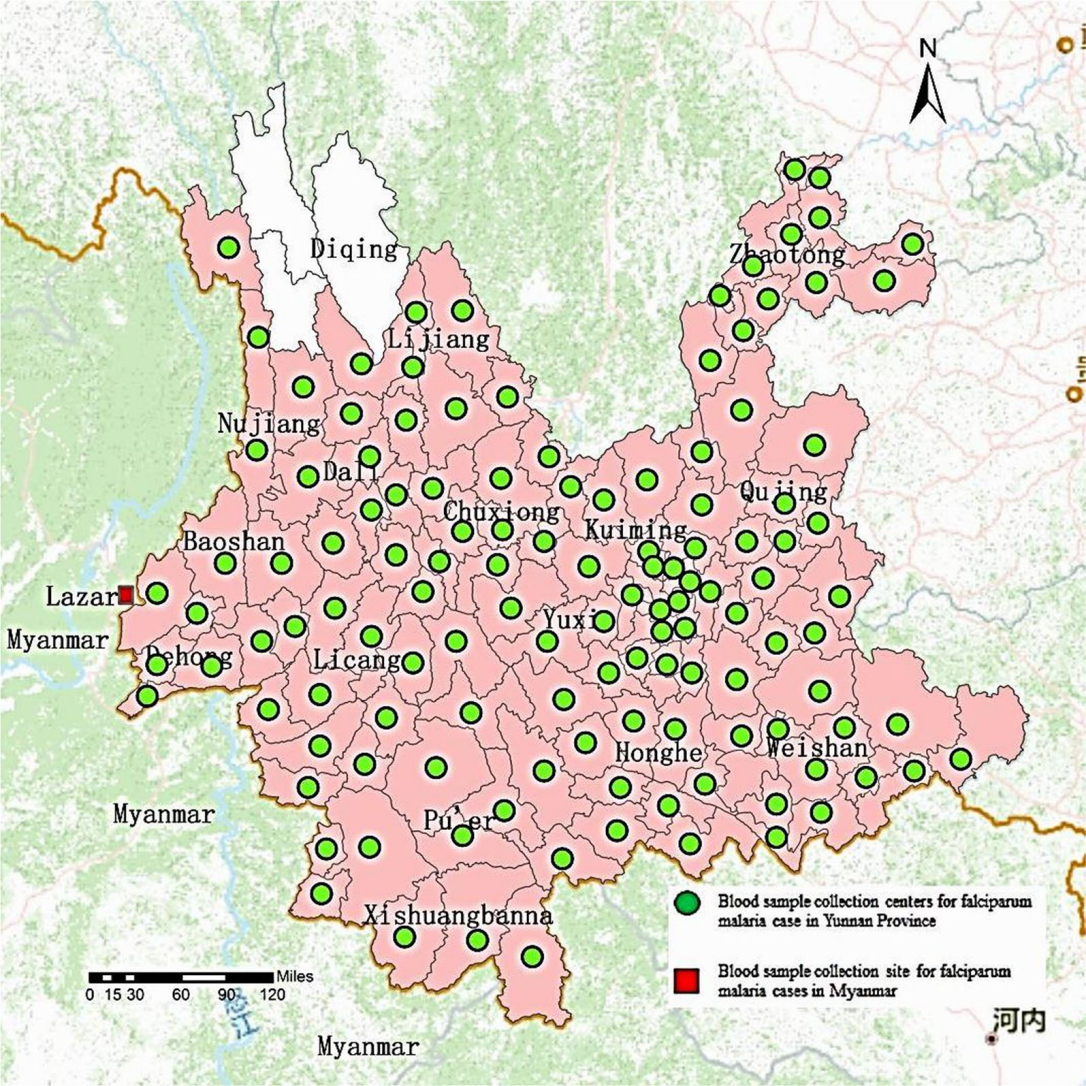
Collection range of *P. falciparum* blood samples from malaria cases in Yunnan Province and in Myanmar. Light red renge indicates falciparum malaria cases found during study period, including Lijiang, Dali, Nujiang, Baoshan, Dehong, Licang, Pu’er, Xishuangbanna, Honghe, Weishan, Yuxi, Qujing, Zhaotong , Kuming and Chuxiong 15 prefectures in Yunnan Province; White range indicates no falciparum malaria cases found during study period, only a prefecture , Diqing in Yunnan Province

A venous blood sample, 0.6 mL, was withdrawn from the reported malaria cases in Yunnan Province from January 2013 to December 2015 and in Myanmar at 0 day before beginning anti-malarial treatment from January 2009 to December 2012 and stored on Whatman 903 filter paper for subsequent use. The sampling points were illustrated in Fig. 1. All the patients signed the informed consent before participation in the study.

### Extraction of *Plasmodium* DNA

*Plasmodium* genomic DNA was extracted from three Whatman 903 filter papers containing the blood samples more than 100 µl, according to the manufacturer’s instructions of DNA Extraction Kit that was purchased from Qiagen (Germany), and was stored at − 20 °C.

### Nested PCR amplification of *Plasmodium falciparum k13* gene

Primers for the propeller domain of the *k13* gene were designed as described previously [9, 37, 43]. The forward and reverse primers for the first round of PCR amplification were 5′-CGGAGTGACCAAATCTGGGA-3′ and 5′-GGGAATCTGGTGGTAACAGC-3′, respectively that amplified 1724435–1726531 bp region in chromosome 13 sequence of *P. falciparum* 3D7 (GenBank Accession Number, CP017003.1), and the product size was expected to be about 2095 bp. The forward and reverse primers for the second round of PCR amplification were 5′-GCCAAGCTGCCATTCATTTG-3′ and 5′-GCCTTGTTGAAAGAAGCAGA-3′, respectively that amplified 1724469–1725317 bp in chromosome 13 (GenBank Accession Number, CP017003.1), where was coding region from 444 to 691 (corresponding 1330–2073 base) in propeller domain of *k13*, and the product size was expected to be about 849 bp. Both first reaction and second PCR reactions contained 2.6 µL DNA template when second reaction the product of the first reaction was used as template, 14 µL of 2× *Taq* PCR mixed system (containing *Taq* enzyme) that was purchased from Qiagen Biotech (Shanghai), 0.7 µL each forward and reverse primers (20 µmol/L), and ddH_2_O for a 25 µL volume. The conditions for the first round PCR reaction were as follows: 95 °C for 2 min, 30 cycles of 95 °C for 30 s, 56 °C for 90 s, and 72 °C for 90 s, and 72 °C for 10 min. The conditions for the second round PCR reaction were as follows: 95 °C for 2 min, 30 cycles of 95 °C for 30 s, 60 °C for 90 s, and 72 °C for 90 s, and 72 °C for 10 min. The amplicons of the second PCR amplification were detected by 1.5% agarose gel electrophoresis, that agarose and DNA standards were procured from Takara Biomedical Technology (Dalian). Then the positive products were sent to Shanghai Meiji Biomedical Technology Co., Ltd. for sequencing using the dideoxy chain-termination method.

### Evolution analysis of *k13* gene polymorphism

DNA sequences from PCR product sequencing were aligned with the reference sequence of *P. falciparum k13* (GenBank Accession Number, PF3D7-1343700) using BLAST module of NCBI after splicing or transformation in the DNAStar Lasergene 7.1 software [37, 38, 44]. The sorted DNA sequences were converted to amino acid sequences using MEGA 5.04 software [37, 38, 44]. The correctness of non-synonymous and synonymous mutations found by DNAStar software was further confirmed by multiple alignments of the DNA sequence using the Clustal X2.1 software [37, 38, 44]. The wild-type amino acids of the 19 loci at 446, 450, 458, 459, 469, 481, 483, 492, 519, 533, 556, 574, 578, 580, 581, 668, 675, and 676 in the propeller domain of the *k13* gene were “FGNSCAFLYGPEPACVEAA” with (GenBank Accession Number, PF3D7-1343700) as the reference, which was considered to be an artemisinin-sensitive sequence of *P. falciparum* isolate [45]. Arlequin 3.01 software was used to analyse the haplotypes and their expected heterozygosity (He) in the gene fragments, gene polymorphism coefficient (H), and genetic differentiation index (Fst) among populations. The Fst was calculated using the molecular variance analysis (AMOVA) with an inspection level of α = 0.05 [37]. The non-synonymous substitution (Ka), synonymous substitution (Ks), and the ratio of these nucleotides was calculated using DnaSP 5.10 software. The Ka/Ks ratio > 1 or < 1 was inferred as positive and negative selection [44], respectively. Network 4.6.0 software was adopted to construct the haplotypes mediatory network of the propeller domain of the *k13* gene [38]. The number and proportion of mutation types in *k13* were counted and analysed using SPSS 21.0 software [37, 38].

### In vivo test of artemisinin resistance

Drugs and treatment protocol was followed according to that by Wang et al. [42], and the curative effect was evaluated using the 28-day in vivo observation recommended by the WHO [46–48]. The cases were followed up on days 1–7, 14, 21, and 28 after artesunate treatment and the observation indexes included recovery time of body temperature and disappearance time of *Plasmodium* in peripheral circulating blood. The artesunate treatment was considered a failure (resistance) if one of the following conditions occurred: (1) patients suffered from parasitaemia and showed signs of danger (or severe malaria) on any day during 1st to 3rd after administration of the drugs. (2) The density of *Plasmodium* on day 2 after medication was higher than that on the day of medication. (3) On day 3 after medication, the patients continued to exhibit parasitaemia, and the axillary temperature was higher than 37.5 °C. (4) On day 3 after medication, the density of *Plasmodium* was > 25% than that on the day of medication. (5) After day 3 of medication, the patients demonstrated parasitaemia and signs of danger (or severe malaria). (6) On any day between 4th and 28th after medication, the patients still demonstrated parasitaemia with an axillary temperature > 37.5 °C (or a history of fever). An unconditional logistic regression model was utilized to estimate the odds ratio (OR) and 95% confidence interval (CI) of mutations in *k13* after the failure of artesunate treatment in falciparum malaria, with an inspection level *α *= 0.05.

## Results

### Samples and nested PCR amplification

**Fig. 2:**
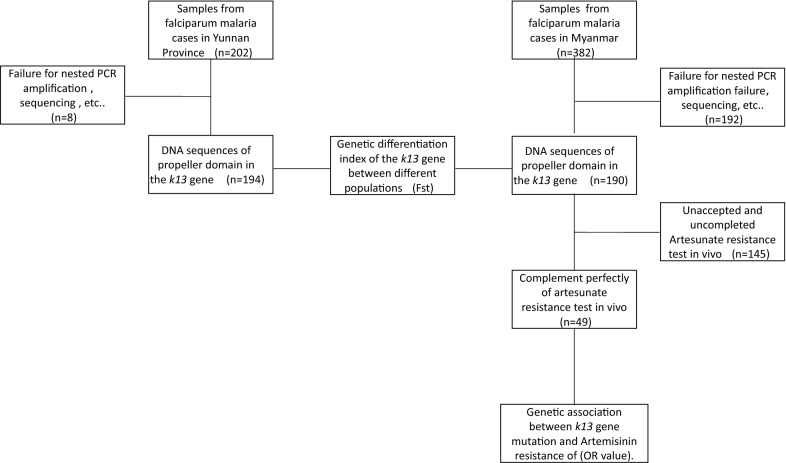
Using flow of falciparum malaria cases’ blood samples in this study

A total of 202 blood samples were collected from falciparum malaria cases in Yunnan province between 2013 and 2015, which included 198 samples that PCR-amplified the propeller domain of the *k13* gene; of these, 98.0% (194/198) samples were successfully sequenced which these samples came respectively from 8 Yunnan indigenous infection cases, 32 infection cases imported from Africa and 154 infection cases imported from Myanmar. A total of 382 blood samples were collected from falciparum malaria cases at 0 day before beginning artesunate treatment in Myanmar between 2009 and 2011, which included 289 samples the positively PCR-amplified the *k13* gene; of these, 190 samples were sequenced successfully in which 49 sequences were collected from the cases who presented artesunate resistance in vivo after the drug treatment full course. The schematic representation of the samples is shown in Fig. 2.

**Fig. 3:**
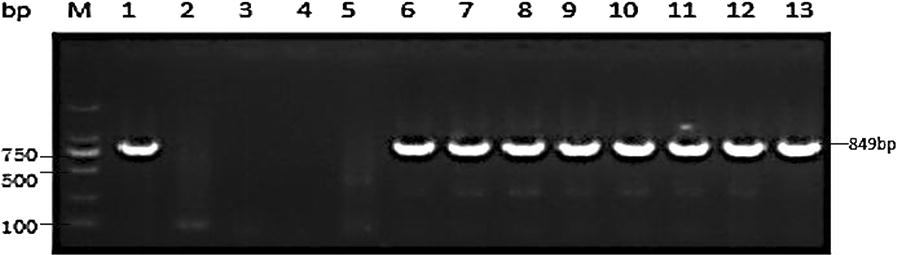
Electrophoresis of nested PCR product of propeller domain in *k13* gene from falciparum malaria cases blood samples. M: DNA marker; 1: *P. falciparum* positive control; 2: *P. vivax* positive control; 3: negative control of the first PCR; 4: negative control of the second PCR; 5: Negative samples; 6–13: positive samples

The PCR amplification products of the *k13* gene in the blood samples of the above two groups are shown in Fig. 3. The electrophoresis showed an 849-bp band of the target gene with a positive amplification rate of 83.4% (487/584).

### Genetic differentiation of *k13* genes in different populations

The DNA sequences of the *k13* gene from 382 cases of falciparum malaria in Yunnan Province and Myanmar presented 23 haplotypes, which expected heterozygosity (He) and gene polymorphism coefficient (H) were 0.6124 and 0.0283, respectively. Moreover, 12 haplotypes in the DNA sequences of cases blood samples diagnosed and reported by Yunnan Province showed expected heterozygosity (He) and gene polymorphism coefficient (H) of 0.0481 and 0.5183, respectively, while 18 haplotypes in the DNA sequences of the cases blood samples in Myanmar showed He and H of 0.0440 and 0.6510, respectively (Table 1).

**Table 1:** Genotypes and their frequencies of propeller domain in the *k13* gene from *P. falciparum* isolates of Yunnan Province and Myanmar

Genotypes and/or gene mutation loci	Haplotypes^C^	Coding single nucleotide polymorphism	Locus code and base substitution^D^	Total number (%)	1. Malaria cases in Yunnan Province No. (%)	2. Malaria cases in Myanmar No. (%)
Sequences of *k13* gene			–	384	194	190
Wild genotype	Hap_1	–	–	202	126	76
Synonymous mutations			–			
G449G	Hap_17	c.1347 T > G	GG***T ***> GG***G***	1 (0.3)	0	1 (0.5)
T451T	Hap_13	c.1353 T > C	TT***T ***> TT***C***	1 (0.3)	1 (0.5)	0
G674G	Hap_22	c.2022 G > A	GG***G ***> GG***A***	1 (0.3)	0	1 (0.5)
Single mutants genotype				–		
F446I*	Hap_2	c.1338 T > A	***T***TT > ***A***TT	130 (33.6)	49 (25.3)	81 (42.6)
G450V*	Hap_15	c.1349 G > T	G***G***A > G***T***A	1 (0.3)	0	1 (0.5)
N458Y*	Hap_7	c.1372 A > T	***A***AT > ***T***AT	3 (0.8)	2 (1.0)	1 (0.5)
S459L*	Hap_9	c.1376 C > T	T***C***G > T***T***G	2 (0.5)	2 (1.0)	0
C469Y*	Hap_10	c.1407 G > A	T***G***C > T***A***C	4 (1.0)	1 (0.5)	3 (1.6)
A481V*	Hap_20	c.1442 C > T	G***C***T > G***T***T	2 (0.5)	0	2 (1.0)
F483S*	Hap_18	c.1448 T > C	T***T***T > T***C***T	1 (0.3)	0	1 (0.5)
L492S*	Hap_19	c.1475 T > C	T***T***A > T***C***A	1 (0.3)	0	1 (0.5)
G533A*	Hap_12	c.1598 G > C	G***G***T > G***C***T	2 (0.5)	1 (0.5)	1 (0.5)
P553L*	Hap_16	c.1658 C > T	C***C***G > C***T***G	3 (0.8)	0	3 (1.6)
E556D*	Hap_5	c.1668 A > T	GA***A ***> GA***T***	3 (0.8)	1 (0.5)	2 (1.0)
P574L*	Hap_8	c.1721 C > T	C***C***T > C***T***T	7 (1.8)	2 (1.1)	5 (2.6)
A578S*	Hap_11	c.1732 G > T	***G***CT > ***T***CT	1 (0.3)	1 (0.5)	0
C580Y*	Hap_14	c.1739 G > A	T***G***T > T***A***T	4 (1.0)	0	4 (2.1)
V581I*	Hap_3	c.1741 G > A	***G***TT > ***A***TT	1 (0.3)	1 (0.5)	0
E668D*	Hap_4	c.2004 G > C	GA***G ***> GA***C***	1 (0.3)	1 (0.5)	0
A675V*	Hap_23	c.2024 C > T	G***C***T > G***T***T	1 (0.3)	0	1 (0.5)
A676D*	Hap_6	c.2027 C > A	G***C***C > G***A***C	11 (2.9)	6 (3.2)	5 (2.6)
Double mutants genotype					–	
Y519K*	Hap_21	c.1855 T > A, c.1857 T > G	***T***A***T ***> ***A***A***G***	1 (0.3)	0	1 (0.5)
G449G/A676D*	Hap_17	c.1337T > G, c.2027 C > A	GG***T ***> GG***G***,G***C***C > G***A***C	Number was the same above synonymous mutations		Yes
Haplotype				23	12	18
Ka/Ks				12.2	16.3	10.9
He				0.6124	0.0481	0.044
H				0.0283	0.5182	0.651
Fst (*P* value)				–	0.0410^A^ (0.0020^B^)	

**Fig.4:**
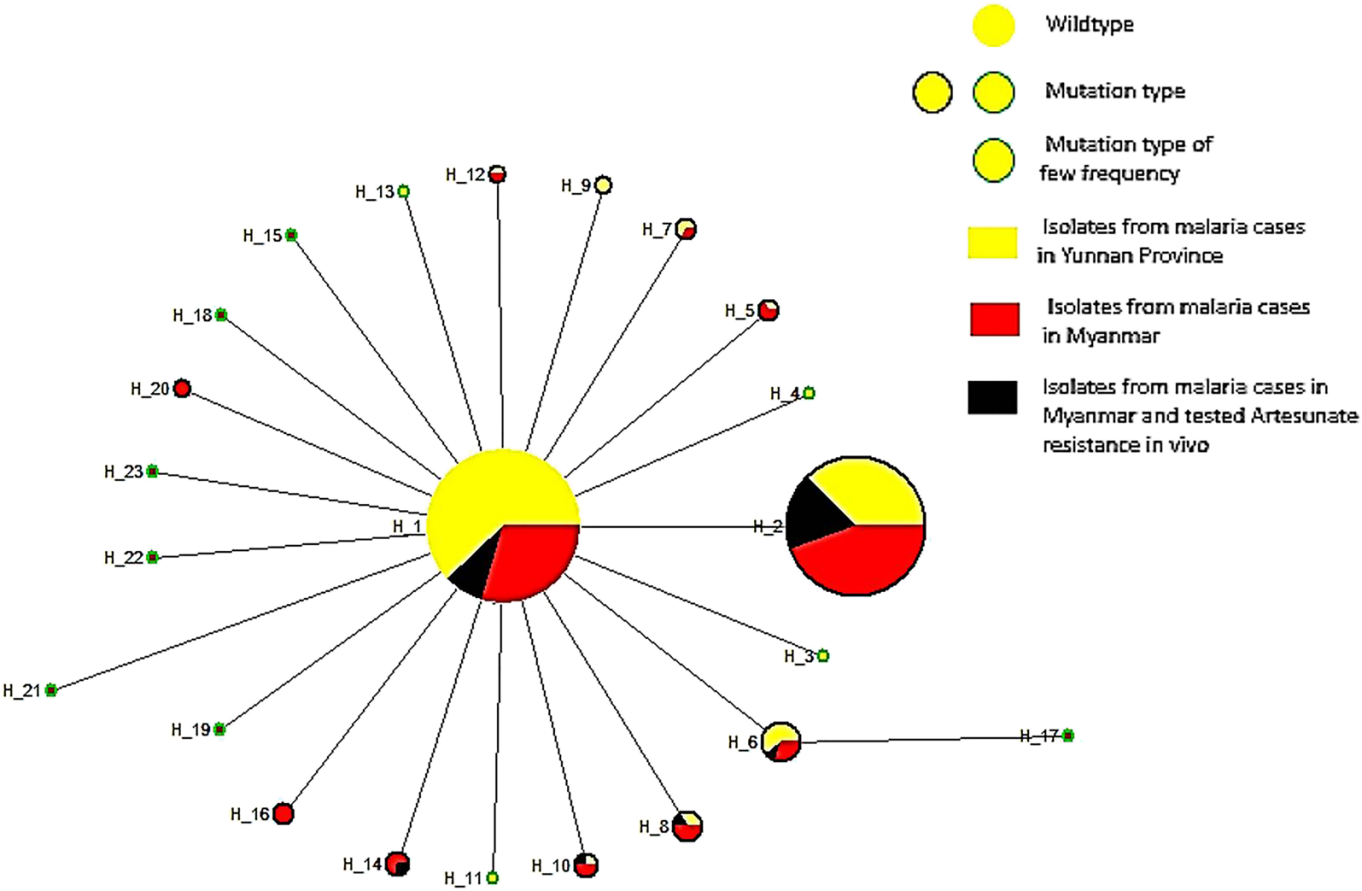
Haplotype network diagram of propeller domain in *k13* gene of *P. falciparum* isolates from malaria cases in Yunnan Province and in Myanmar. The size of the circle was proportional to the number of isolates showing particular haplotypes; the lines represent evolutional steps connecting haplotypes

* Letters in bold indicate mutated amino acids; A: Group contribution ratio between group 1 and group 2 of Fst; B: *P*<0.05; C: The names of Haplotypes were the same of Fig. 4; D: The substituted bases highlighted with italic and bold in every code

A total of 3 synonymous, 19 non-synonymous mutations, and one wild-type were detected in the 23 haplotypes, wherein the third bases of code were substituted in the synonymous mutation loci of 449, 451 and 674; on the contrary, the substituted proportions of the first base, second base and third base of code were 26.3% (5/19), 63.2% (12/19) and 10.5% (2/19), respectively, in the non-synonymous loci of 446, 450, 458, 459, 469, 481, 483, 492, 519, 533, 553, 556, 574, 578, 580, 581, 668, 675, and 676. Especially,in the 519 locus, the double mutation was found due to substitute simultaneously at the first and third base (Table 1). Among the 194 sequences from falciparum malaria case samples in Yunnan Province, there were single mutations at 11 loci of 446, 458, 459, 469, 533, 556, 574, 578, 581, 675, and 676 with different mutation rates. Among the 190 sequences from falciparum malaria case samples in Myanmar, single mutations were detected at 13 loci of 446, 450, 458, 469, 481, 483, 492, 533, 553, 556, 574, 580, 675 and double mutations be detected at 2 loci of 519, 676 (Table 1).

The Fst between the *P. falciparum* population in Yunnan Province and that in Myanmar was 0.0410 (P < 0.05). The intra-population mutation accounted for 95.9%, while the inter-population mutation accounted for 4.1% (Table 1), thereby indicating that the intra-population differentiation was greater than the inter-population differentiation. In addition, the haplotypes mediatory network exhibited a similar evolutionary trend of DNA sequences of the *k13* gene in both populations. The stellar network started with the wild-type “FGNSCAFLYGPEPACVEAA” haplotype (H_1), and the mutated haplotypes were one-step evolution except for the synonymous mutation haplotype (H_17) (Fig. 4). A majority of the mutants had a single mutation (H_2) at the locus 446 (Fig. 4); however, the incidence of the low-frequency mutation (3.7%, 7/190) was higher in isolates from Myanmar than that from the Yunnan Province (2.1%, 4/194). Moreover, the mutants at locus 676 included non-synonymous mutation haplotype (H_6) and double mutation haplotype (H_17). The ratios of Ka/Ks of *k13* in both the Yunnan and Myanmar populations were > 1 (16.3, 10.9, respectively), which indicated that the DNA sequence undergoes positive diversified selection.

### Correlation between gene mutant and artemisinin resistance

Among the 49 cases of falciparum malaria patients in Myanmar, who completed the in vivo test of artemisinin resistance and the follow-up, the clinical success rate was 93.9% (46/49), and the failure rate was 6.1% (3/49). The blood samples from the cases that completed the in vivo test were paired for polymorphism analysis of the *k13* gene. Consequently, five types of mutations were detected in *k13* in 28 cases, with a mutation rate of 57.1% (28/49), and the composition ratios were 82.1% (23/28) for F446I, 7.1% (2/28) for C469Y, and 3.6% (1/28) for A676D, N458Y, and P574L. The correlation between the failure of artesunate treatment and mutations at loci of *k13* was shown in Table 2, which indicated that the risk of artemisinin resistance in the *k13* gene with mutation at locus 446 of the propeller domain was 1.640-fold that of the wild-type gene (95% CI 1.284–2.095), and the risk was approximately 1.840-fold when the five types of mutation loci were combined (95% CI 1.412–2.398).

**Table 2:** The genetic association between the *k13* mutations of *P. falciparum* isolates and artemisinin resistance from falciparum malaria cases in Myanmar

Mutation loci	Frequency	OR value	95% CI	
			Lower	Upper
F446I or N458Y or C469Y or P574L or A676D	28	1.840	1.412	2.398
F446I	23	1.640	1.284	2.095

## Discussion

The present study was designed to detect the locus mutations at the propeller domain of artemisinin-resistant *Pf*K13 protein in *Plasmodium* in 194 cases of falciparum malaria in Yunnan Province from January 2013 to December 2015 [9, 37, and 43]. A total of 11 single-locus non-synonymous mutations F446I, N458Y, S459L, C469Y, G533A, E556D, P574L, A578S, V581I, E668D, and A676D were detected in the 444–709 aa region at the C-terminal of *Pf*K13 protein. Among these loci, S459L and E668D were discovered recently [14, 16, 19–21, 43, 49–52], while the highest mutation rate of 21.1% at locus 446 (Table 1) was lower than that in the blood sample from Myanmar as reported by Tun et al. [14, 19]. Moreover, this mutation rate was lower than 73.2%, 27.2%, and 70.8% detected in blood samples from China-Myanmar border areas as reported by Huang et al. [21] and Wang et al. [20, 52], which might be associated with the heterogeneity of samples used in various studies. In a majority of the previous studies on the artemisinin resistance markers from blood samples of patients with falciparum malaria in the China-Myanmar border region, the clinical efficacy of anti-malarial drugs was evaluated [21, 45, 52, 53]. Nevertheless, the blood samples in the present study were collected from falciparum malaria cases from all the geographical areas of the Yunnan Province for consecutive 3 years, without any limitations on the density of *Plasmodium* and clinical manifestations. Therefore, the samples were continuous and systematic, allowing the monitoring of artemisinin resistance markers similar to the routine conditions in Yunnan Province.

Notably, the mutants that were not detected at loci 493, 539, 543, and 580 were considered to be closely related to the phenotype of artemisinin resistance in 194 blood samples collected from falciparum malaria cases in Yunnan Province [54, 55], and the multivariate mutations described by Taylor et al. [49] and Huang et al. [51] were not found in each mutation locus. This phenomenon suggested that the locus mutation associated with artemisinin resistance might occur when the *k13* gene mutation is accumulated. A total of 15 non-synonymous mutation loci F446I, G450V, N458Y, C469Y, A481V, F483S, L492S, Y519K, G533A, P553L, E556D, P574L, C580Y, A675V, and A676D were detected in the propeller domain in the *Pf*K13 protein of *P. falciparum* in blood samples collected from 190 falciparum malaria cases in Myanmar. Among them, G450V, Y519K, and A675V were discovered recently [14, 16, 19–21, 43, 49–52]. The double mutation consisting of synonymous mutations at locus 449 and non-synonymous mutations at locus 676 was found in one case (Fig. 4). Moreover, 2.1% of the samples presented mutations at locus 580 [54, 55] that was closely related to the phenotype of artemisinin resistance (Table 1). In addition, the mutation rate at locus 446 was 42.6%, which was higher than that in the isolates from cases in the Yunnan Province, thereby indicating pronounced hyper-mutation of the falciparum malaria cases in Myanmar [56, 57]. In addition, a lower mutation rate in *k13* in the falciparum malaria cases in the isolates in Yunnan might be attributed to the 15.1% African isolates in the samples. These African isolates are mainly derived from Angola, Cameroon, Congo, Guinea, Nigeria, Tanzania, Mali, Ethiopia, Chad, and Gabon that are still considered as areas with a lower pressure of artemisinin drugs than that in Southeast Asia [11, 12, 14, 58].

Fst is a critical indicator of the degree of differentiation between subpopulations and populations, which can be used to quantify the genetic relationship between different populations. The value of Fst ranges from 0 to 1, and it refers to a similar genotype in the random mating and a unique genotype in complete isolation, respectively, when used for comparison between the populations [59]. In the present study, Fst was used to evaluate the degree of differentiation of *k13* between the population of *P. falciparum* isolates in Yunnan and Myanmar. The results demonstrated that although the type of mutations and the types and number of haplotypes in the *k13* gene of two *P. falciparum* from falciparum malaria cases in Yunnan and Myanmar were different (Table 1), the genetic differentiation coefficient between the two groups was small (Fst = 0.0410, *P *< 0.05). Furthermore, the intra-population and the inter-population variation accounted for 95.9% and 4.1%, respectively. Hence, in the present study, similar genetic backgrounds were detected in the populations of *P. falciparum* isolates from cases in Yunnan and Myanmar. Therefore, the degree of risk of artemisinin resistance in the *k13* gene mutation obtained in the isolates from Myanmar (Table 2) could also be reported in the falciparum malaria cases in Yunnan. This phenomenon indicated that the risks of artemisinin treatment failure in Yunnan cases infected with *P. falciparum* with 446I mutations or that in any locus of 446I, 469Y, 676D, 458Y, and 574L in the *k13* gene were 1.640-fold (95% CI 1.284–2.095) and 1.840-fold (95% CI 1.412–2.398) of the cases infected with wild-type *P. falciparum*, respectively. Unlike the evaluation of the genetic association of artemisinin resistance in the China-Myanmar border region reported by Huang et al. [21] and Wang et al. [53], the results of the current study could be utilized to deduce the hazards of *k13* mutation in the Yunnan Province. Nonetheless, no correlation was detected between the *k13* gene mutation and artemisinin resistance in the isolates in Yunnan cases. However, the polymorphism mutation loci, especially the mutation at locus F446I in *k13*can be used as a molecular marker for monitoring the artemisinin resistance in *P. falciparum* in Yunnan Province.

Recently, the stellar layout of the mediatory network of haplotypes has been considered as evidence of population expansion [60–63]. Herein, both the evolution networks of haplotypes in the *k13* gene in *P. falciparum* isolates from Yunnan and Myanmar cases were stellar, and the low-frequency haplotypes accounted for a large proportion in the population. These results demonstrated a continuous expansion of the *P. falciparum* population in the two groups, which is affected by the external environment screening. Together with the Ka/Ks ratio > 1 in both groups (Table 1), the “positive diversified selection” from the two populations indicated that the *P. falciparum* escapes the pressure of artemisinin.

The non-parametric correlation analysis is the simplest associative analysis method used in the case–control study for the direct comparison between the two groups with respect to the alleles and gene frequencies of genetic markers. A significant correlation between diseases with some alleles can guide the development of causal relationship study, and ultimately could provide the direction for finding the genetic causes of disease susceptibility. Nevertheless, the present study has limitations. First, the sample size was small for the genetic association study, and the degree of risk of *k13* mutation needs to be elucidated further. Second, the subjects undergoing a phenotype test are foreign ethnicity in Myanmar, which might cause race-related genetic heterogeneity in Yunnan cases. In addition, the area for the phenotypic study, Lazan Myanmar, shows a high prevalence of malaria. Therefore, the use of microcosmic evaluation indicators obtained in this study should be employed cautiously in different falciparum malaria endemic areas. The expansion of the sample size of the homogenous study and the relevant systematic analysis of the genetic relationship between Artemisinin resistance phenotype and *k13* gene mutation in *P. falciparum* are imperative for future investigations.

In this study, the successful rates of PCR amplification and sequencing were not near 100%, which may be related to the different preservation durations of and the different cryopreservation conditions of blood samples during last many years. In addition, whether the density of plasmodium and the concentration of genomic DNA extracted from falciparum malaria cases blood effect on the efficiency of PCR amplification and the successful rate of sequencing of PCR products? They will be verified in another study.

Finally, the original intention using these blood samples for research should be explained further. In China, diagnosis, reporting and management of the malaria cases are carried out in administrative regions, such as in a province and a county. In the management measures to malaria cases, the identification of the Yunnan indigenous infection cases or cases imported is mainly to facilitate statistics of malaria elimination evaluation indicators and this identification has no special guiding role whether selecting some control and protection measures in appearing malaria epidemic situation area. On the contrary, because the potential epidemic hazards both indigenous and imported cases are the same, so the epidemic interdiction measures adopted are almost as same as comprehensive and systematic, for example, these measures must be carried out such as screening *Plasmodium* infection for health residents, protection of susceptible population from malarial interruption, reducing vectors density for malaria transmission. Therefore, with the *Plasmodium* isolates of falciparum malaria cases reported by Yunnan Province as the research samples, it was not only helpful to reflect the continuity and integrity of management for falciparum malaria cases in Yunnan Province, but also necessary to understand the biological characteristics of the special malaria case isolates population for Yunnan Province. In previous studies, some genes of the *Plasmodium* population of falciparum malaria cases isolates found in Yunnan Province, which included almost 80% of the infection cases imported from Myanmar and few other parts from Yunnan indigenous infection cases and infection cases imported from Africa, had only existed a very weak genetic differentiation between these and pure Myanmar cases isolates populations [37, 38, 64]. This study also showed that there was no significant differentiation of *k13* gene between the two populations. These results suggest that the characteristic of the population imported from Myanmar are confounded by a small amount of Yunnan indigenous isolates or isolates imported from Africa in all of Yunnan falciparum cases isolates.

## Conclusions

By using the method of population genetics, the slight genetic differentiation has been found between the *Plasmodium* isolates from falciparum malaria cases in Yunnan Province and those in Myanmar. Although two groups of isolates are discovered from different sites, they still belong to the same geographical population. It is reasonable to take artemisinin resistance characterization of *Plasmodium* population in Myanmar as the contrast to the artemisinin sensitivity status of *Plasmodium* population in Yunnan Province. As a result, based on the molecular epidemiological investigation on the propeller domain mutation of *k13* gene in *Plasmodium* isolates from falciparum malaria cases in Yunnan Province and the determination of the artemisinin resistance on falciparum malaria cases in Myanmar, 12 mutations loci including 446 locus and other 11 loci in *k13* of *Plasmodium* isolated from falciparum malaria cases in Yunnan Province were found to be positively genetic correlated with artemisinin resistance. This provides a basis for further monitoring the artemisinin resistance molecular markers of *Plasmodium* in Yunnan Province. It also provides a useful experience for these areas that how to carry out anti-malarial drug resistance phenotype observation when facing the shortage of clinical volunteers.

## References

[1] Mok S, Ashley EA, Ferreira PE, Zhu L, Lin Z, Yeo T (2005). Population transcriptomics of human malaria parasites reveals the mechanism of artemisinin resistance. Science.

[2] Tu Y (2011). The discovery of artemisinin (qinghaosu) and gifts from Chinese medicine. Nat Med.

[3] Miller LH, Su X (2011). Artemisinin: discovery from the Chinese herbal garden. Cell.

[4] Zhang YL, Pan WQ (2015). Research progress on artemisinin resistance in *Plasmodium falciparum*. Chin J Parasitol Parasit Dis..

[5] Noedl H, Se Y, Schaecher K, Smith BL, Socheat D, Fukuda MM (2008). Evidence of artemisinin-resistant malaria in western Cambodia. N Engl J Med.

[6] Amaratunga C, Sreng S, Suon S, Phelps ES, Stepniewska K, Lim P (2012). Artemisinin-resistant *Plasmodium falciparum* in Pursat province, western Cambodia: a parasite clearance rate study. Lancet Infect Dis..

[7] Phyo AP, Nkhoma S, Stepniewska K, Ashley EA, Nair S, McGready R (2012). Emergence of artemisinin-resistant malaria on the western border of Thailand: a longitudinal study. Lancet.

[8] Kyaw MP, Nyunt MH, Chit K, Aye MM, Aye KH, Aye MM (2013). Reduced susceptibility of *Plasmodium falciparum* to artesunate in southern Myanmar. PLoS ONE.

[9] Ashley EA, Dhorda M, Fairhurst RM, Amaratunga C, Lim P, Suon S (2014). Spread of artemisinin resistance in *Plasmodium falciparum* malaria. N Engl J Med.

[10] Dondorp AM, Nosten F, Yi P, Das D, Phyo AP, Tarning J (2009). Artemisinin resistance in *Plasmodium falciparum* malaria. N Engl J Med.

[11] Wootton JC, Feng X, Ferdig MT, Cooper RA, Mu J, Baruch DI (2002). Genetic diversity and chloroquine selective sweeps in *Plasmodium falciparum*. Nature.

[12] Gething PW, Patil AP, Smith DL, Guerra CA, Elyazar IR, Johnston GL (2011). A new world malaria map: *Plasmodium falciparum* endemicity in 2010. Malar J..

[13] Zhou SS, Wang Y, Feng W, Tang LH (2009). Malaria situation in the People’s Republic of China in 2008. Chin J Parasitol Parasit Dis..

[14] Tun KM, Imwong M, Lwin KM, Win AA, Hlaing TM, Hlaing T (2015). Spread of artemisinin-resistant *Plasmodium falciparum* in Myanmar: across-sectional survey of the K13 molecular marker. Lancet Infect Dis..

[15] Mbengue A, Bhattacharjee S, Pandharkar T, Liu H, Estiu G, Stahelin RV (2015). A molecular mechanism of artemisinin resistance in *Plasmodium falciparum* malaria. Nature.

[16] Ariey F, Witkowski B, Amaratunga C, Beghain J, Langlois AC, Khim N (2014). A molecular marker of artemisinin-resistant *Plasmodium falciparum* malaria. Nature.

[17] Takala-Harrison S, Clark TG, Jacob CG, Cummings MP, Miotto O, Dondorp AM (2013). Genetic loci associated with delayed clearance of *Plasmodium falciparum* following artemisinin treatment in Southeast Asia. Proc Natl Acad Sci USA.

[18] Adams J, Kelso R, Cooley L (2000). The kelch repeat superfamily of proteins: propellers of cell function. Trends Cell Biol.

[19] Tun KM, Jeeyapant A, Imwong M, Thein M, Aung SS, Hlaing TM (2016). Parasite clearance rates in upper Myanmar indicates a distinctive artemisinin resistance phenotype: a therapeutic efficacy study. Malar J..

[20] Wang Z, Shrestha S, Li X, Miao J, Yuan L, Cabrera M (2015). Prevalence of K13-propeller polymorphisms in *Plasmodium falciparum* from China-Myanmar border in 2007–2012. Malar J..

[21] Huang F, Takala-Harrison S, Jacob CG, Liu H, Sun X, Yang H (2015). A single mutation in K13 predominates in Southern China and is associated with delayed clearance of *Plasmodium falciparum* following artemisinin treatment. J Infect Dis.

[22] Che LG, Huang KG, Yang HL (1984). [Determination of the susceptibility for *Plasmodium falciparum* isolates to pyridine](in Chinese). Chin J Parasitol Parasit Dis..

[23] Yang HL, Yang PF, Dong Y, Che LG, He H, Liu DQ (1994). Longitudinal surveillance of chloroquine resistance of *Plasmodium falciparum* after cessation of medication in south Yunnan. Chin J Parasitol Parasit Dis..

[24] Yang HL, Li CF, Yang YM, Yang PF, Yang R, Nie RH (2008). Longitudinal monitoring in vivo sensitivity of *Plasmodium falciparum* to chloroquine in the Southeast of Yunnan, China. J Pathog Biol..

[25] Che LG, Chen W, Yang HL (1986). Growth and decline of chloroquine-resistance *Plasmodium falciparum* prevalence in Yunnan Province. Chin J Epidemiol..

[26] Li HX, Chen GW, Yang YC, Jiang H (2008). Malaria situation in Yunnan Province during 2001–2005. Chin J Parasitol Parasit Dis..

[27] Che LG (1981). Clinical study on the treatment effect of new antimalarial drug, “224”, on 65 falciparum malaria cases. Med Pharm Yunnan..

[28] Wang TY, Tang BZ, Xu RC, Li Y, Zhao XZ, Xu XY (1981). Clinical study on the treatment efficacy of artemisinin derivative, “224” and “242”, for falciparum malaria. Acta Academiae Medicine Kunming..

[29] Wang TY (1981). Clinical study on the treatment efficacy of artemisinin for malaria. Intermediate Med J..

[30] Guangdong College of traditional Chinese Medicine (1979). Analysis of the treatment efficacy on 40 cerebral malaria cases with artemisinin. Guangdong Health Epidemic Prevention..

[31] Guo XB, Fu LC, Jian HX, Li GQ, Wang WL, Cai DF (1986). Report of clinical efficacy on 32 cases dangerous malaria treated with artemisinin suppository. Acta Academiae Medicine Guangzhou..

[32] Guangdong College of traditional Chinese Medicine (1979). Analysis of the clinical efficacy of artemisinin on 36 cases with chloroquine-resistance malaria. Guangdong Health Epidemic Prevention..

[33] Liu DQ, Liu RJ, Zhang CY, Cai XZ, Tang X, Yang HL (1996). Present status of the sensitivity of *Plasmodium falciparum* to antimalarials in China. Chin J Parasitol Parasit Dis..

[34] Liu DQ, Feng XP, Yang HL, Lin SG, Chen WJ, Yang PF (2005). Fluctuation in the resistance of *Plasmodium falciparum* to chloroquine in China. Chin J Parasitol Parasit Dis..

[35] Yang HL, Li XL, Gao BH, Yang PF, Zhang ZY (2009). Surveillance of *Plasmodium falciparum* susceptibility to seven antimalarials, including artemether in the western part of the Sino-Myanmar border area. J Pathog Biol..

[36] Zhu YJ, Chen MN, Xu YC, Mao XH, Deng Y, Dong Y (2016). Polymorphism of 72-76 coding sequence within exon 2 region of Pfcrt in Yunnan Province. Chin J Parasitol Parasit Dis..

[37] Sun AM, Dong Y, Chen MN, Xu YC, Deng Y, Mao XH (2016). Polymorphism analysis of *Plasmodium falciparum* K13 gene Kelch domain associated with resistance to artemisinin in Yunnan Province. Chin J Parasitol Parasit Dis..

[38] Dong Y, Sun AM, Deng Y, Chen MN, Xu YC, Mao XH (2017). Analysis on co-mutation of chloroquine-resistant gene and artemisinin-resistant gene in *Plasmodium falciparum* in Yunnan Province. Chin J Parasitol Parasit Dis..

[39] Zhang L, Feng J, Zhang SS, Xia ZG, Zhou SS (2016). Malaria situation in the People’s Republic of China in 2015. J Pathog Biol..

[40] Department of Health of China. [Diagnostic criteria for malaria (WS 259-2006). People’s Republic of China health industry standard. 2006. 17597—2006 **(in Chinese)**.

[41] Dong Y, Mao XH, Chen MN, Deng Y, Wang J, Xu YC (2015). [Quality analysis of laboratory malaria diagnosis in Yunnan Province during 2012–2014. Chin J Parasitol Parasit Dis..

[42] Wang J, Xu YC, Sun XD, Li CF, Zhang PW, Lin ZR (2016). An analysis of the diagnosis and treatment of quarrian malaria among febrile patients in northern Myanmar. J Pathog Biol..

[43] Nyunt MH, Hlaing T, Oo HW, Tin-Oo LL, Phway HP, Wang B (2015). Molecular assessment of artemisinin-resistance markers, polymorphisms in the K13 propeller and a multidrug-resistance gene, in eastern and western border areas of Myanmar. Clin Infect Dis.

[44] Dong Y, Deng Y, Xu YC, Chen MN, Mao XH, Sun AM (2018). Analysis of genes associated with antifolate drug resistance in *Plasmodium vivax* from different infection sources. Chin J Parasitol Parasit Dis..

[45] Feng J, Zhou D, Lin Y, Xiao H, Yan H, Xia Z (2015). Amplification of pfmdr1, pfcrt, pvmdr1, and K13 propeller polymorphisms associated with *Plasmodium falciparum* and *Plasmodium vivax* isolates from the China-Myanmar border. Antimicrob Agents Chemother.

[46] WHO. Assessment of therapeutic efficacy of antimalarial drugs for uncomplicated falciparum malaria in areas of intense transmission. Geneva: World Health Organization; 1996. WHO/MAL/96.1077.

[47] WHO. Monitoring antimalarial drug resistance. In: *Plasmodium falciparum* therapeutic efficacy test methods. Geneva: World Health Organization; 2001. http://researchonline.lshtm.ac.uk/4979/.

[48] Sun XD, Zhang ZX, Wang J, Deng Y, Yang YM, Lasi J (2011). Therapeutic efficacy and safety of compound dihydroartemisinin/piperaquine for uncomplicated *Plasmodium falciparum* infection in Laiza City of Myanmar bordering on China. Chin J Parasitol Parasit Dis..

[49] Taylor SM, Parobek CM, DeConti DK, Kayentao K, Coulibaly SO, Greenwood BM (2015). Absence of putative artemisinin resistance mutations among *Plasmodium falciparum* in sub-Saharan Africa: a molecular epidemiologic study. J Infect Dis.

[50] Thuy-Nhien N, Tuyen NK, Tong NT, Vy NT, Thanh NV, Van HT (2017). K13 Propeller mutations in *Plasmodium falciparum* populations in regions of malaria endemicity in Vietnam from 2009 to 2016. Antimicrob Agents Chemother.

[51] Huang B, Deng C, Yang T, Xue L, Wang Q, Huang S (2015). Polymorphisms of the artemisinin resistant marker (K13) in *Plasmodium falciparum* parasite populations of Grande Comore Island 10 years after artemisinin combination therapy. Parasit Vectors..

[52] Wang Z, Wang Y, Cabrera M, Zhang Y, Gupta B, Wu Y (2015). Artemisinin resistance at the China-Myanmar border and association with mutations in the K13 propeller gene. Antimicrob Agents Chemother.

[53] Wang Y, Yang Z, Yuan L, Zhou G, Parker D, Lee MC (2015). Clinical efficacy of dihydroartemisinin-piperaquine for the treatment of uncomplicated *Plasmodium falciparum* malaria at the China-Myanmar border. Am J Trop Med Hyg.

[54] Amato R, Lim P, Miotto O, Amaratunga C, Dek D, Pearson RD (2017). Genetic markers associated with dihydroartemisinin-piperaquine failure in *Plasmodium falciparum* malaria in Cambodia: a genotype-phenotype association study. Lancet Infect Dis..

[55] Straimer J, Gnädig NF, Witkowski B, Amaratunga C, Duru V, Ramadani AP (2015). Drug resistance K13-propeller mutations confer artemisinin resistance in *Plasmodium falciparum* clinical isolates. Science..

[56] Lee AH, Fidock DA (2016). Evidence of a mild mutation phenotype in Cambodian *Plasmodium falciparum* malaria parasites. PLoS ONE.

[57] Ljolje D, Dimbu PR, Kelley J, Goldman I, Nace D, Macaia A (2018). Prevalence of molecular markers of artemisinin and lumefantrine resistance among patients with uncomplicated *Plasmodium falciparum* malaria in three provinces in Angola, 2015. Malar J..

[58] Ghansah A, Amenga-Etego L, Amambua-Ngwa A, Andagalu B, Apinjoh T, Bouyou-Akotet M (2014). Monitoring parasite diversity for malaria elimination in sub-Saharan Africa. Science.

[59] Ye R, Hu D, Zhang Y, Huang Y, Sun X, Wang J (2016). Distinctive origin of artemisinin resistant *Plasmodium falciparum* on the China-Myanmar border. Sci Rep..

[60] Wu F, Hu W, Wang C, Guo B, Mo C (2016). Comparative analysis on genetic diversity between culture perch and wild population. Acta Hydrobiol Sin.

[61] Miao M, Yang Z, Patch H, Huang Y, Escalante AA, Cui L (2012). *Plasmodium vivax* populations revisited: mitochondrial genomes of temperate strains in Asia suggest ancient population expansion. BMC Evol Biol.

[62] Taylor JE, Pacheco MA, Bacon DJ, Beg MA, Machado RL, Fairhurst RM (2013). The evolutionary history of *Plasmodium vivax* as inferred from mitochondrial genomes: parasite genetic diversity in the Americas. Mol Biol Evol.

[63] Gupta B, Reddy BP, Fan Q, Yan G, Sirichaisinthop J, Sattabongkot J (2015). Molecular evolution of PvMSP3αblock II in *Plasmodium vivax* from diverse geographic origins. PLoS ONE.

[64] Dong Y, Sun AM, Chen MN, Xu YC, Mao XH, Deng Y (2016). Analysis of polymorphism of *Pfhrp*2 gene in *Plasmodium falciparum* from falciparum malaria patients in Yunnan Province. Chin J Schistosomiasis Control..

